# Genome Similarities between Human-Derived and Mink-Derived SARS-CoV-2 Make Mink a Potential Reservoir of the Virus

**DOI:** 10.3390/vaccines10081352

**Published:** 2022-08-19

**Authors:** Mohammad Khalid, Anas Alshishani, Yousef Al-ebini

**Affiliations:** 1 Department of Pharmaceutics, College of Pharmacy, King Khalid University, Asir-Abha 61421, Saudi Arabia; 2 Faculty of Pharmacy, Zarqa University, Zarqa 13110, Jordan

**Keywords:** mutation, SARS-CoV-2, nucleotide sequences, mink, zoonosis

## Abstract

SARS-CoV-2 has RNA as the genome, which makes the virus more prone to mutations. Occasionally, mutations help a virus to cross the species barrier. SARS-CoV-2 infections in humans and minks (*Neovison vison*) are examples of zoonotic spillover. Many studies on the mutational analysis of human-derived SARS-CoV-2 have been published, but insight into the mink-derived SARS-CoV-2 genome of mutations is still required. Here, we performed a mutation analysis of the mink-derived SARS-CoV-2 genome sequences. We analyzed all available full-length mink-derived SARS-CoV-2 genome sequences on GISAID (214 genome sequences from the Netherlands and 133 genome sequences from Denmark). We found a striking resemblance between human-derived and mink-derived SARS-CoV-2. Our study showed that mutation patterns in the SARS-CoV-2 genome samples from the Netherlands and Denmark were different. Out of the 201 mutations we found, only 13 mutations were shared by the Netherlands’ and Denmark’s mink-derived samples. We found that six mutations were prevalent in the mink-derived SARS-CoV-2 genomes, and these six mutations are also known to be prevalent in human-derived SARS-CoV-2 variants. Our study reveals that the G27948T mutation in SARS-CoV-2 leads to truncation of ORF8, which was also reported in human-derived SARS-CoV-2, thus indicating that the virus can replicate without the full-length ORF8. These resemblances between mink-derived and human-derived SARS-CoV-2 enable the virus to cross the species barrier and suggest mink a potential reservoir for the virus.

## 1. Introduction

The COVID-19 pandemic is caused by SARS-CoV-2. The human transmission of SARS-CoV-2 is a zoonotic spillover as a consequence of mutational changes in the viral genome [1]; these changes are an ongoing process, and they keep producing variants of the virus. The ΔH69/V70 variant of the virus was reported to be more transmissible and deadly [2,3]. The ΔH69/V70 variant of SARS-CoV-2 has a deletion of two amino acids in the spike (S) protein of the virus. The S protein is a crown-like structure, which is why the virus was named the coronavirus [4]. The S protein of the virus uses the angiotensin-converting enzyme 2 (ACE-2) as a receptor to enter the host cells [4,5,6]. Apparently, if the ACE-2 receptor of different hosts has an affinity toward the S protein of the virus, the virus is capable of crossing the interspecies barrier. This statement itself is very threatening and could have tremendous implications.

Minks (*Neovison vison*) in Dutch farms with symptoms of respiratory illnesses and higher mortality rates were found to be SARS-CoV-2 positive [7]. Munnink et al. (2021) reported that people working on these farms were also infected with SARS-CoV-2 [8], suggesting that the virus can transmit back and forth between minks and humans. Similar cases have also been reported from Danish mink farms [9,10,11], which suggests that the infected mink could be a reservoir and/or a potential source for the transmission of the virus to humans.

European farmers are the highest producers of mink fur from 6000 farms across Europe. They account for 63% of the world’s production of mink fur. Denmark is the leading producer of mink fur and accounts for around 28% of world production [12].

SARS-CoV-2 has a 29.9-kilobases, positive-sense, single-stranded RNA genome (+ssRNA) [13]. The RNA makes the virus relatively more susceptible to mutational changes [14]. Many reports regarding the mutation patterns in human-derived SARS-CoV-2 have been already published [15,16,17,18], but we do not have sufficient information about the mutation patterns of the mink-derived SARS-CoV-2 samples.

Our goal was to analyze the mink-derived SARS-CoV-2 genome sequences and investigate the mutation pattern in the genome. In this study, we examined all mink-derived full-length SARS-CoV-2 genome sequences available in GISAID [19].

## 2. Methods

### 2.1. Mink-Derived SARS-CoV-2 Genome Sequences

In the present study, we analyzed all available mink-derived full-length SARS-CoV-2 genome sequences in GISAID [19]. As of 4 November 2020, 347 sequences were available (set: November 2020); out of those 347 sequences, 214 were from mink farms in the Netherlands and 133 were from mink farms in Denmark. Once we found a pattern of mutations, further analyses for specific mutations was performed on 755 genome sequences available on 22 January 2021 (set: January 2021). Out of these 755 SARS-CoV-2 genome sequences, 454 were from Denmark, 278 were from the Netherlands, 12 were from Poland, 7 were from Lithuania, and 4 were from Canada.

### 2.2. Mutational Analysis of the Mink-Derived SARS-CoV-2 Genome Sequences

To analyze the November 2020 dataset of mink-derived full-length SARS-CoV-2 genome sequences, we followed the method described elsewhere [20]; briefly, a software tool for fragmenting nucleotide sequences, Fragmentation Tool_Version-1.0, was used to fragment the genome sequences. It was possible to align the 3000-base-long SARS-CoV-2 genome fragments in the MultiAlin online tool [21] with the reference strain of the virus (accession number NC_045512.2) [22]. Each fragment was set to contain 100-base-long overlapping sequences to ensure that the fragments did not miss part of the genome sequence. We considered variations from the reference strain as mutations if variations were repeated in more than one instance. The mutations found in the mink-derived SARS-CoV-2 genome in the November 2020 dataset were further analyzed in the January 2021 dataset.

## 3. Results

### 3.1. Pipeline for the Mutation Analysis

**Chart 1:**
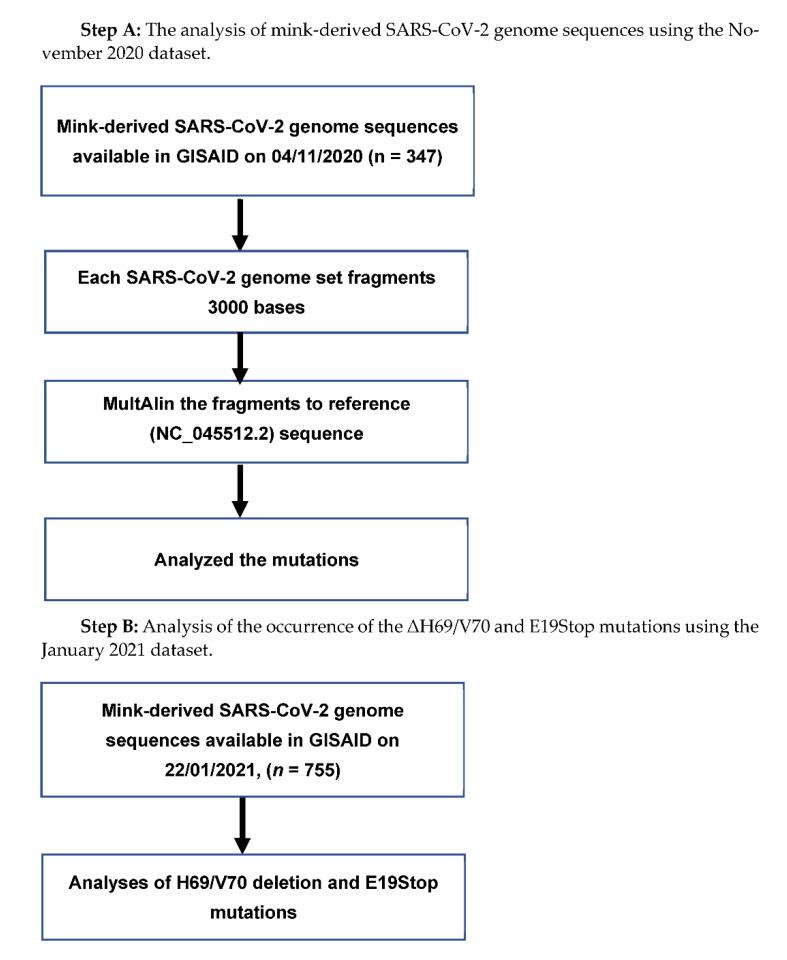
Flow chart of the mink-derived SARS-CoV-2 genome sequence analysis.

Pipeline for the Mutation Analysis is follow Chart 1:

### 3.2. SARS-CoV-2 Hosts Range

Table 1 provides the details of the SARS-CoV-2 genome sequences derived from various infected hosts and submitted to GISAID. These sequences were available as of 22 January 2021. Mink (*Neovison vison*) seems to be highly susceptible to SARS-CoV-2 infection; the mink-derived samples contributed the 755 genome sequences. SARS-CoV-2 was also found to have infected several other animals, e.g., cat, pangolin, dog, tiger, lion, bat monkey, and mouse, as shown in Table 1.

### 3.3. The Phylogenetic Tree of SARS-CoV-2 Genomes Derived from Various Hosts

**Figure 1:**
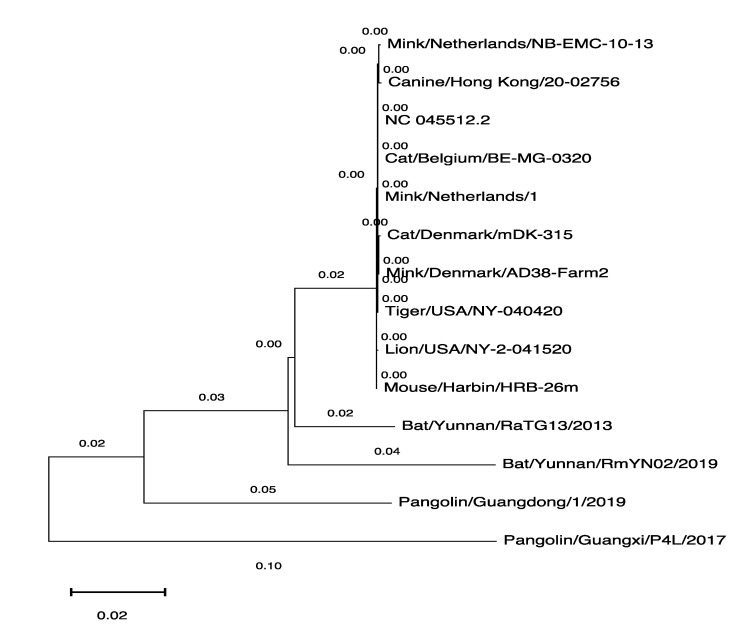
Phylogenetic tree of the SARS-CoV-2 genomes derived from various hosts. The MEGAX software tool was used to generate the phylogenetic tree.

We wanted to analyze similarities between the SARS-CoV-2 genome sequences derived from the various hosts shown in Table 1, so we used the MEGAX software tool [23] to generate the phylogenetic tree with the genomic sequences available in GISAID. We used one SARS-CoV-2 genomic sequence derived from every host as a representative. The human-derived SARS-CoV-2 genome sequence was used as a reference (NC_45512.2) to establish the phylogenetic relationship between these sequences (Figure 1).

### 3.4. The Mutations in SARS-CoV-2 Genome Derived from the Netherlands and Denmark Mink Farms

**Figure 2:**
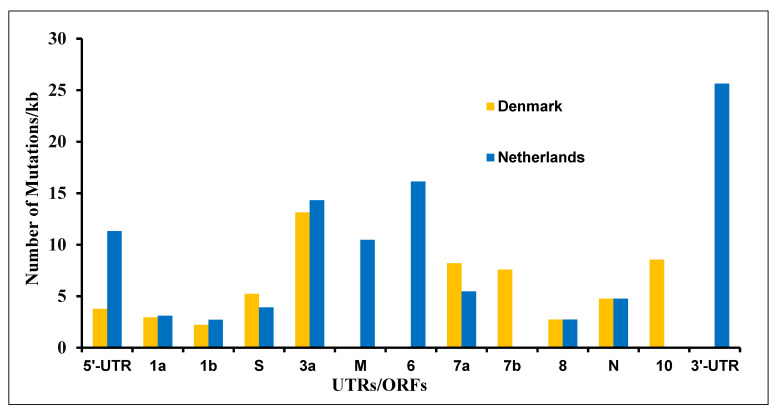
Mutations in various ORFs in the SARS-CoV-2 genome sequences derived from the Netherlands and Denmark.

As shown in Table 1, the mink (*Neovison vison*)-derived SARS-CoV-2 genome sequences were the second highest, and mink was found to be highly susceptible to viral infection among the tested animals. We were curious to find mutation patterns in the mink-derived SARS-CoV-2 genomes. We analyzed 347 (November 2020 set; 214 from the Netherlands and 133 from Denmark) SARS-CoV-2 genome sequences available as of 4 November 2020. We found 201 mutations in the SARS-CoV-2 genome. These mutations were found to occur in every UTR/ORF of the mink-derived SARS-CoV-2 genomes except ORF-E (Figure 2 and Supplementary Table S1). The viral sequences derived from Dutch and Danish mink farms showed different mutation patterns (Supplementary Table S1). Out of 201 mutations, only 12 mutations (C241T, T3037C, A5421G, ATA6510deletion, G11083T, C14408T, C15656T, A22920T, A23403G, C25936T, G26062T, and C26078T) were common among the Netherlands- and Denmark-derived samples. Interestingly, out of these, five mutations (C241T, T3037C, G11083T, C14408T, and A23403G) were also reported in human-derived SARS-CoV-2 genomes [20].

### 3.5. The ΔH69/V70 and Several Other Mutations Were Prevalent in Mink-Derived SARS-CoV-2 Genome

We found a total of 201 mutations, and 18 of those mutations were found to be prevalent in SARS-CoV-2 (occurred in more than 50% of samples); see Table 2 and Supplementary Table S1. Out of these 18 mutations, eight mutations (C241T, T3037C, ATA6510deletion, C14408T, C15656T, A22920T, A23403G, and C25936T) were found to be prevalent in both the Denmark- and Netherlands-derived samples. The ACATGT21766deletion mutation, which translates to H69/V70 deletion in the S protein, occurred in 84.2% of samples derived from Denmark. None of the Netherlands’ samples were positive for the ACATGT21766deletion (H69/V70 deletion) mutation; see Table 2 and Supplementary Table S1. The A23403G mutation, which translates to the D614G mutation in the S protein, was shown to be dominant in human-derived SARS-CoV-2 [24] and was also found to be prevalent in Denmark’s (100%) and the Netherlands’ (89.3%) mink-derived SARS-CoV-2 samples; see Table 2 and Supplementary Table S1.

### 3.6. Mutations at Amino Acid Level

**Figure 3:**
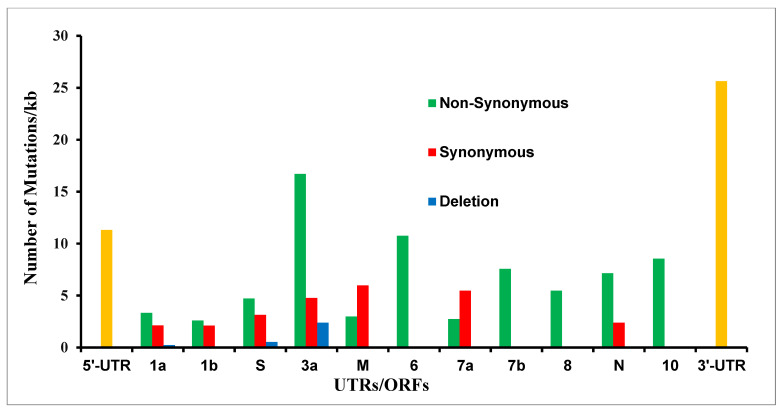
The synonymous, non-synonymous, and deletion mutations at the amino acid level in the mink-derived SARS-CoV-2 genome from Netherlands and Denmark samples. The UTRs (untranslated regions) are shown in yellow.

We translated all 201 SARS-CoV-2 genomic mutations and found that the majority of them (122) are non-synonymous mutations that lead to amino acid changes; meanwhile, ORF-M and 7a had more synonymous than non-synonymous mutations (4 out of 6 and 2 out of 3, respectively); see Figure 3 and Supplementary Table S1. The five mutations, which do not code for any proteins, lay between ORFs, i.e., ATATTAGTTTTTCTGTTTGG26487 deletion (between ORF-3a and M), G27390A (between ORF-6 and 7a), CTTATT27697deletion (between ORF-7a and 7b), T29685C, and T29726 deletion (between ORF-10 and 3′-UTR); see Supplementary Table S1.

### 3.7. The Frame-Shift and Nonsense Mutations

During the mutational analysis, we found TTAATCCAGTA26159deletion (which leads to frame-shift mutations) in 1.9% of the Netherlands’ samples; see Supplementary Table S2. The mutation was found to lie at position 256 of ORF-3a, which is 276 amino acids long, leads to a change in amino acid sequences after position 256. We looked at the frame-shift mutation in the January 2021 dataset, and we did not find any other sample with this mutation.

We also found the G27948T mutation in 2.3% of Denmark-derived samples; this mutation lies at ORF-8 and is translated to a stop codon after amino acid 18 in ORF, which is 122 amino acids long. We also analyzed nonsense mutation in the January 2021 dataset, and we found that 15 samples, all derived from Denmark, had this mutation, which was shown to lead to truncated ORF-8 expression; see Supplementary Table S3.

## 4. Discussions

The binding capability of the SARS-CoV-2 S protein to the ACE-2 receptor of a host’s cells makes these cells susceptible to viral entry, which determines the host range and tissue tropism of SARS-CoV-2. Table 1 reveals the broad mammalian hosts’ range of SARS-CoV-2. However, for productive viral replication, mere viral entry is not enough; compatibilities of the host’s cellular machinery are also essential, and this could be a reason for a handful of reports of the SARS-CoV-2 infection of domesticated animals. Perhaps host factors are restricting the virus’ ability to replicate and transmit in those mammals. These plausible factors need to be explored for their therapeutic applications in human counterparts.

The first transmission of the virus to humans in the Wuhan seafood market was said to be from bat/pangolin [25], but we have only a few SARS-CoV-2 genomes derived from these suspected animals (as shown in Table 1). On the other hand, the 755 genomic sequences derived from the mink (*Neovison vison*) and the majority of minks found infected in many farms in the Netherlands and Denmark [7,8,9,10,11] indicate that minks are highly susceptible to viral infection. The phylogenetic tree shown in Figure 1 illustrates the similarities in the genomic sequences of the virus; the human-derived viral sequence is more similar to mink-derived viral sequences than bat- or pangolin-derived viral sequences. The close resemblance between human and mink-derived SARS-CoV-2 enables the virus to transmit between humans and minks, which is how the people working in mink farms were infected [8] and makes minks a potential SARS-CoV-2 reservoir. This reservoir may have the capability to produce variants of varying infectivity, which has been identified in humans [26].

In this study, we found 201 mutations from the analysis of 347 samples (214 from the Netherlands and 133 from Denmark) available in GISAID. The mutation patterns in these genomic sequences were very different (Supplementary Table S1). Out of the 201 mutations found, 113 were from Netherlands-derived samples and 101 were from Denmark-derived samples. Of those 201 mutations, 18 mutations were shown to prevalent in Netherlands-derived samples, Denmark-derived samples, or both (Table 2 and Supplementary Table S1). Remarkably, from these 18 mutations, 6 (C241T, P314L, D614G, S3194L, RG203KR, and H69/V70 deletion) were also shown to be prevalent in human-derived SARS-CoV-2 genome samples. The C241T mutation in 5′-UTR is known to have a global frequency of more than 95% [27]. The famous D614G mutation in the spike protein, which is known to increase infectivity [24], and the co-occurrence of D614G and P314L mutations in ORF1b of human-derived SARS-CoV-2 samples [28] were also found in mink-derived samples in our study. The S194L mutation in the nucleocapsid protein of human-derived samples was found to be a significant occurrence in the mortality group [29] also found in mink-derived samples. The RG203KR mutations in the nucleocapsid may play role in the antibody neutralization reactions [30] found in human-derived SARS-CoV-2 samples; our study also revealed RG203KR mutations in mink-derived SARS-CoV-2 from Denmark. Lastly, the H69/V70 deletion in the Alpha variant was previously found to increase infectivity [31] and was also found to be prevalent in mink-derived samples from Denmark studied here.

We found the TTAATCCAGTA26159 deletion, which leads to frame-shift mutation after amino acid 255 in ORF-3a (which is 276 amino-acid-long protein), in four samples derived from the Netherlands; see Supplementary Tables S1 and S2. We analyzed the January 2021 dataset, but we did not find the deletion mutation in any sample except the four samples mentioned in Supplementary Table S2.

Another mutation G27948T in ORF-8, which generates a stop codon after 18 amino acids, was found in two samples derived from Denmark. We analyzed the January 2021 dataset for the mutation and found that 15 samples, all from Denmark, had the nonsense mutation; see Supplementary Tables S1 and S3. A similar mutation has also been reported in human-derived SARS-CoV-2 samples in many studies [20,32,33], which shows that SARS-CoV-2 can replicate without the full-length ORF-8 protein. Studies have shown that ORF-8 plays a role in MHC-1 expression [32,33], and a 29-nucleotide deletion in the SARS-CoV ORF8ab truncation was reported to attenuate the viral replication [34]; however, the functional role of ORF-8 in SARS-CoV-2 requires further investigation.

In this study, all mutation analyses of the SARS-CoV-2 genomes were performed in reference to a Wuhan-derived sequence (NC_22452.2), which was used to establish close resemblance in the genome sequences derived from human and mink hosts. Although the mutation patterns observed in the mink-derived samples from the Netherlands and Denmark were different, different patterns have also been found in human-derived SARS-CoV-2 genome sequences [20].

On the other hand, some common mutations were found to be prevalent in the SARS-CoV-2 genomes derived from human and mink hosts. These findings further validate the similarities of the SARS-CoV-2 genomes derived from human and mink hosts. The similarities between human and mink-derived SARS-CoV-2 also open paths for animal study; the mink could be a promising animal model for vaccine and therapeutic studies.

## 5. Conclusions

The SARS-CoV-2 infection of humans is a zoonotic spillover facilitated by interactions between viral proteins and the host’s cellular machinery. The mutation capability not only helps the virus to escape from vaccines and antiviral drugs but also provides unlimited possibilities to cross the species’ barriers. The susceptibility of minks to SARS-CoV-2 infection is another example of zoonotic spillover. We do not have reason to believe that the zoonotic spillover will not happen again. It is just a matter of time before mutant variants of the virus will find compatible hosts. As reports have suggested [8], the virus is transmitted between humans and minks, which would also be possible for any new host the virus may find.

## Figures and Tables

**Table 1:** SARS-CoV-2 hosts.

S. No.	Name	Sequences
1	Human (*Homo sapiens*)	3,240,911
2	Mink (*Neovison vison*)	994
3	Cat (*Felis catus*)	70
4	Lion (*Panthera leo*)	37
5	Dog (*Canis lupus familiaries*	28
6	Pangolin (*Manis javanica*)	19
7	Tiger (*Panthera tigris jacksoni*)	13
8	Otter (*Aonyx cinereus*)	5
9	Mouse (*Mus musculus*)	4
10	Bat (*Rhinolophus malayanus*)	4
11	Bat (*Rhinolophus shameli*)	2
12	Bat (*Rhinolophus affinis*)	1
13	Monkey (*Chlorocebus sabaeus*)	1
14	Pangolin (*Manis pentadactyla*)	1

The SARS-CoV-2 complete genome sequences derived from various hosts, available in GISAID as of 21 December 2021.

**Table 2:** Prevalent mutations in mink-derived SARS-CoV-2 genome.

S. No.	Mutation	Amino Acid	ORFs	Denmark %	Netherlands %
1	C241T	N/A	5′-UTR	100.0	90.7
2	TTA516---	M84 deletion	1a	83.5	0.0
3	C1380T	A372V	1a	0.0	50.9
4	T3037C	Silent	1a	100.0	77.6
5	C5144T	Silent	1a	84.2	0.0
6	ATA6510---	S2082 deletion	1a	84.2	16.0
7	C11776T	Silent	1a	84.2	0.0
8	G14274A	Silent	1b	0.0	51.4
9	C14408T	P314L	1b	100.0	89.3
10	C15656T	T730I	1b	93.2	5.7
11	ACATGT21766------	H69/V70 deletion	S	84.2	0.0
12	A22920T	Y453F	S	90.2	19.8
13	A23403G	D614G	S	100.0	89.3
14	A24862G	Silent	S	0.0	51.4
15	C25936T	H182Y	3a	93.2	43.9
16	G28854T	S194L	N	84.2	0.0
17	GGG28881AAC	RG203KR	N	97.7	0.0
18	T29726-	-	10/3′-UTR	83.5	0.0

## Data Availability

Not applicable.

## Supplementary Materials

The following supporting information can be downloaded at: https://www.mdpi.com/article/10.3390/vaccines10081352/s1, Table S1. Mutations in mink-derived SARS-CoV-2 genome from Netherlands’ and Denmark’s samples; Table S2. The sample details of TTAATCCAGTA26159deletion (which leads to frame-shift mutations) in mink-derived SARS-CoV-2 genome from Netherlands’ samples; Table S3. The details of the mink-derived SARS-CoV-2 genome with non-sense mutation in ORF-8 at position 19.

## Abbreviations

GISAID	Global initiative on sharing all influenza data
NCBI	National Center for Biotechnology Information
NSP6	Non-structural protein 6
ORF-8	Open Reading Frame-8
UTRs	Untranslated regions
